# Treatment of Tumors by Electrolysis

**Published:** 1883-10

**Authors:** 


### Treatment of Tumors by Electrolysis.

Neftel has returned to this method of treating malignant tumors, destroying them at a single operation. A platinum anode is plunged perpendicularly into the tumor down to its presumed point of implantation, and from three to five cathodes placed on the periphery of the tumor. The current is then closed and rapidly carried to its greatest power (30 to 60 elements). The position of the cathodes is changed every five minutes, so as to cover every part of the tumor. The operation lasts about an hour. The tumor becomes livid, gray, and finally black. There is a very slight general, and local reaction. In two or three days the part operated upon becomes cold, and after some discharge finally comes away *en bloc*, leaving a denuded surface which is soon covered by healthy granulations. Neftel has also treated benign tumors by this method, though they do not require such energetic treatment as those of the malignant type. The conclusions which he draws are:

1. Electrolysis is an antiseptic method, and as such may be combined with the ordinary methods of operation.

2. It is preferable to any other method in the treatment of malignant tumors.

3. Malignant tumors should be entirely destroyed by the operation, and at a single seance. In benign tumors it is sufficient to establish a retrograde metamorphosis.—*Virchow's Archiv.*

